# mTOR complex-2 stimulates acetyl-CoA and *de novo* lipogenesis through ATP citrate lyase in HER2/PIK3CA-hyperactive breast cancer

**DOI:** 10.18632/oncotarget.8279

**Published:** 2016-03-22

**Authors:** Yaqing Chen, Jianchang Qian, Qun He, Hui Zhao, Lourdes Toral-Barza, Celine Shi, Xuesai Zhang, Jiang Wu, Ker Yu

**Affiliations:** ^1^ Department of Pharmacology, Fudan University School of Pharmacy, Shanghai, China; ^2^ Oncology Research, Pfizer Pharmaceuticals, Pearl River, NY, USA

**Keywords:** mTORC2, ATP citrate lyase, lipid synthesis, breast cancer, cancer metabolism

## Abstract

The mechanistic target of rapamycin (mTOR) is a major regulator of cell growth and is frequently dysregulated in cancer. While mTOR complex-1 (mTORC1) is a validated cancer target, the role of mTOR complex-2 (mTORC2) remains less defined. Here, we reveal mTORC2 as a critical regulator of breast cancer metabolism. We showed that hyperphosphorylation in ATP citrate lyase (ACL) occurs frequently in human breast tumors and correlates well with HER2+ and/or PIK3CA-mutant (HER2+/PIK3CAmut) status in breast tumor cell lines. In HER2+/PIK3CAmut cells, mTORC2 controls Ser-455 phosphorylation of ACL thereby promoting acetyl-CoA production, de novo lipogenesis and mitochondrial physiology, all of which were inhibited by an mTORC1/mTORC2 kinase inhibitor (mTOR-KI) or cellular depletion of mTORC2 or ACL. mTOR-KI but not rapamycin blocked the IGF-1-induced ACL phosphorylation and glucose to lipid conversion. Depletion of mTORC2 but not mTORC1 specifically inhibited the ACL-dependent acetyl-CoA production. In the HER2+/PIK3CAmut MDA361, MDA453, BT-474 and T47D cells, depletion of mTORC2 or ACL led to growth inhibition and mitochondrial hyperpolarization, which were partially rescued by an alternate source of acetyl-CoA. These same changes were not apparent in mTORC2- or ACL-depleted HER2-/PIK3CAwt MDA231 and HCC1806 cells, highlighting a differential dependence of mTORC2-ACL for survival in these two cell types. Moreover, ACL Ser-455 mutants S455E (phosphomimetic) and S455A (non-phosphorylatable) each increased or decreased, respectively, the acetyl-CoA production, mitochondrial homeostasis and survival in ACL-depleted MDA453 cells. These studies define a new and rapamycin-resistant mechanism of mTORC2-ACL in lipogenesis and acetyl-CoA biology and provide a rationale for targeting of mTORC1 and mTORC2 in HER2+/PIK3CAmut breast cancer.

## INTRODUCTION

The mechanistic target of rapamycin (mTOR) acts through two multiprotein complexes, mTOR complex-1 (mTORC1) and mTOR complex-2 (mTORC2), coordinates signals from growth factors, nutrients and energetics. mTOR functions both in parallel and downstream of the PI3K/AKT signaling pathway that is frequently dysregulated in cancer and a subject of intense discovery research [1, 2]. Moreover, activating-mutations within mTOR itself exist in multiple cancer types and may predict therapeutic response to mTOR-targeted therapy [3, 4]. While clinical use of mTORC1 inhibitor rapalog therapy (e.g. temsirolimus, everolimus) validated mTOR as a cancer target, the effectiveness of these drugs may be limited due to resistance to mTORC2 [5, 6]. Accordingly, a new generation mTOR kinase inhibitors (mTOR-KIs) capable of targeting both mTORC1 and mTORC2 have emerged with several of these agents in early stage of patient trials [7–12]. In preclinical setting, mTOR-KIs have demonstrated distinct anticancer pharmacology from those of rapalogs and elicited deeper and broader spectrum suppression of tumor cell growth and induction of apoptosis in molecular subtypes of tumors. At lower end of pharmacological dose range, mTOR-KIs tend to act similarly as rapalogs in that both inhibit cellular proliferation yet attenuate process of aging [13–15]. It is thought that targeting of mTORC1 and mTORC2 with mTOR-KIs will facilitate further dissection of molecular mechanism of mTOR complexes in cancer and a variety of age-related diseases.

mTOR plays an important role in pathogenesis and therapy resistance in breast cancer [16, 17]. As such, the mTORC1 inhibitor everolimus is already marketed for adjuvant therapy in advanced hormone receptor (HR)-positive breast cancer [18, 19]. Clinical studies with rapalogs in HER2-mutant, therapy-resistant patients are underway [16, 20]. Moreover, investigating efficacy of mTOR-KIs in metastatic breast cancer patients are reported (e.g. NCT02216786, NCT02049957). To facilitate these clinical efforts, there is a need to elucidate the new, rapalog-resistant mechanism of mTOR and to characterize molecular subtypes and underlying response biomarkers that are relevant for mTOR-targeted treatments.

Cancer cells feature aerobic glycolysis because it not only satisfies energy production but also generates cellular building-blocks including those for de novo lipid biosynthesis [21, 22]. The constitutive PI3K/AKT signaling is sufficient to promote growth factor-independent glycolysis and de novo lipogenesis [23, 24]. In breast cancer, an elevated basal glycolysis is associated with oncogenic HER2- or estrogen receptor (ER) status [25]; however, the molecular aspects of the oncogenic control in these process remains poorly understood.

We sought to investigate novel role of mTOR in glucose to lipid metabolism in breast cancer, particularly those governed by mTORC2, a key component of HER2, PI3K pathway and is resistant to rapalog therapy. We performed phosphoproteomic analysis of MDA361, a HER2+/PIK3CAmut breast tumor line, treated without or with a highly selective mTOR-KI WYE-125132 (WYE-132) [8]. Herein, we report the identification and characterization of Ser-455 in ATP citrate lyase (ACL) as a molecular target of dysregulated mTORC2 signaling pathway in HER2+/PIK3CAmut breast cancer cells. ACL converts mitochondrially derived citrate to cytosolic lipogenic precursor acetyl-CoA, thereby serving as a key enzyme of de novo lipid synthesis in these cells. Pharmacologic or genetic inactivation of mTORC2 in HER2+/PIK3CAmut cells, but not in HER2-/PIK3CAwt cells, leads to a rapid loss in Ser-455 phosphorylation of ACL. The mTORC2-dependent phosphorylation of ACL plays an important role in de novo lipid synthesis, acetyl-CoA homeostasis, mitochondrial physiology and increased survival. We suggest that down-regulation of mTORC2-ACL axis reflects an important mechanistic aspect in mTOR-targeted cancer therapy.

## RESULTS

### Identification of ACL as an mTOR regulated phosphoprotein

**Figure 1:**
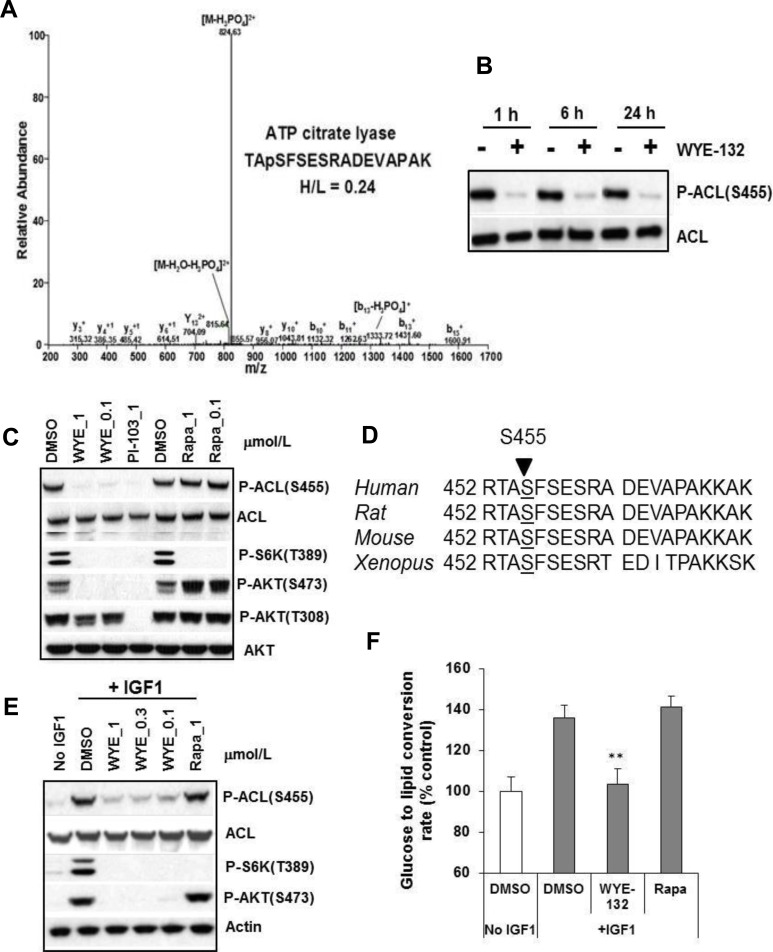
ACL is an mTOR regulated phosphoprotein **A.** MS/MS spectra of ACL phosphopeptide identified by SILAC. The sequence of a tryptic peptide matched to human ACL and the SILAC ratio (heavy-labeled/light-labeled (H/L)) for ACL peptide is shown for the corresponding spectra. **B.** and **C.** MDA361 cells were treated with 1 μmol/L WYE-132 for the indicated times (B) or with various inhibitors for 24 h (C) followed by immunoblotting. **D.** DNA sequence alignment of human, rat, mouse and xenopus ACL gene. **E.** HEK293 cells were serum-depleted overnight, treated with inhibitors for 30 min, stimulated with 100 ng/mL IGF-1 followed by immunoblotting. **F.** HEK293 cells were grown in medium with 1% FBS overnight, treated with DMSO, 1 μmol/L WYE-132 or 1 μmol/L rapamycin for 2 h. The cells were then stimulated for 2 h with 100 ng/mL IGF-1 and ^14^C-glucose, and analyzed for de novo lipid synthesis as described in Methods. Statistical analysis: **, *p* < 0.01.

The SILAC-based phosphoproteomic analysis of MDA361 cells treated without or with a highly selective mTOR-KI WYE-132, yielded a peptide that corresponded to human ACL amino acids 452 to 467. Intensity ratio of WYE-132-treated “heavy”-labeled *versus* the control “light”-labeled phosphopeptides (H/L) for Ser-455-containing peptide was 0.24, indicating a 76% inhibition due to WYE-132 treatment (Figure 1A). We next performed immunoblotting of MDA361 cells employing a phospho-specific P-ACL(S455) antibody. WYE-132 demonstrated a rapid ( < / = 1 h) and sustained (>/ = 24 h) suppression of P-ACL(S455) without affecting total ACL (Figure 1B). Interestingly, P-ACL(S455) was not inhibited by rapamycin and correlated with mTORC2 biomarker P-AKT(S473) and was independent of the classical PI3K biomarker P-AKT(T308) (Figure 1C). Amino acid sequence alignment found that Ser-455 lies in a region that is completely conserved in ACL protein of human, rat, mouse and xenopus (Figure 1D). Because ACL produces cytosolic lipogenic precursor acetyl-CoA, we explored whether mTOR regulates ACL in insulin-like growth factor-1 (IGF-1)-stimulated de novo lipid synthesis. As a major activator of mTOR, IGF-1 induced a rapid ACL Ser-455 phosphorylation and robust glucose to lipid conversion in HEK293 cells, both of which were completely or substantially inhibited by 1 μmol/L WYE-132 but not by 1 μmol/L rapamycin (Figure 1E, 1F) indicating a rapamycin-resistant role of mTOR in this process. Taken together, these observations identify ACL Ser-455 as a molecular target of mTOR in regulating de novo lipid synthesis.

### ACL Ser-455 phosphorylation is widely elevated in breast cancer clinical specimen and cell lines correlating HER2/PIK3CA-hyperactivation

**Figure 2:**
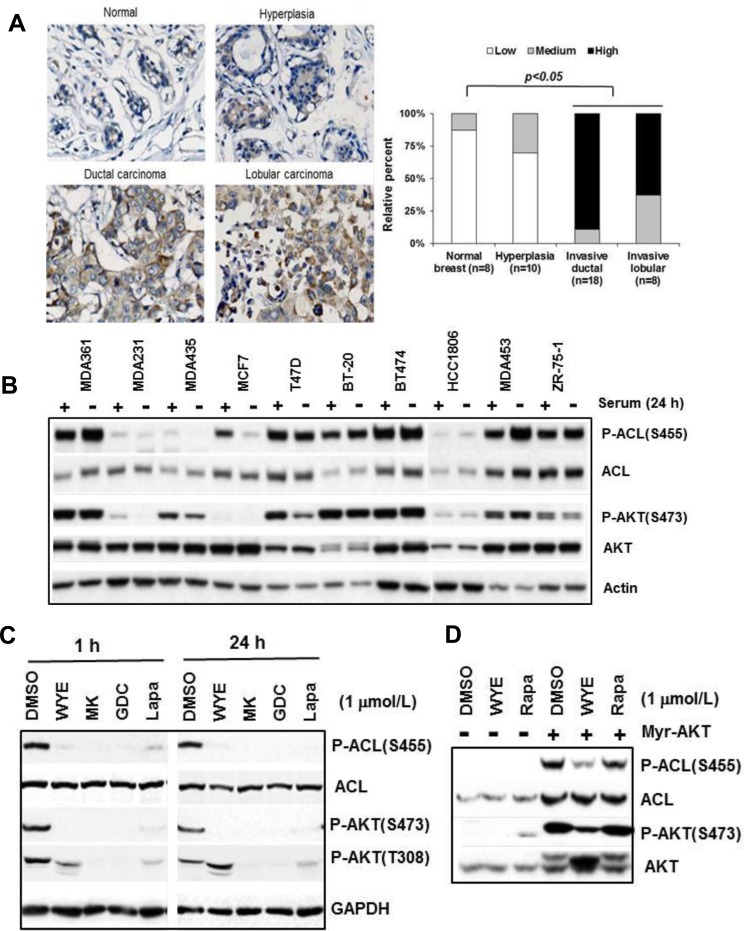
ACL Ser-455 phosphorylation is widely elevated in breast cancer **A.** Tissue array with normal breast (*n* = 8), hyperplasia (*n* = 10), invasive ductal (*n* = 18) and invasive lobular (*n* = 8) tissue samples were subjected to IHC with a phospho-ACL antibody as described in Methods. Representative images are shown (left) and staining results are summarized (right). **B.** Breast tumor cell lines were grown in 10-cm culture dish. Exponentially proliferating cells were fed for 24 h with medium with or without 10% serum. Total cell lysates were subjected to immunoblotting. **C.** MDA453 cells were treated with the indicated inhibitors followed by immunoblotting. **D.** Rat2 and Rat2-myr-AKT cells were treated inhibitors for 24 h followed by immuoblotting.

To explore the relevance of ACL phosphorylation in breast cancer, we performed immunohistochemistry (IHC) on normal- and breast tumor tissues. We first validated the antibody specificity in cultured cells treated with DMSO or WYE-132, in which a positive staining was greatly diminished upon WYE-132 treatment (Figure S1A). P-ACL IHC analysis of tissue array with normal breast (*n* = 8), hyperplasia (*n* = 10), invasive ductal carcinoma (*n* = 18) and invasive lobular carcinoma (*n* = 8) revealed a general trend for low staining in normal breast, a noticeably increased staining in hyperplasia and the highest P-ACL staining in invasive ductal and lobular carcinoma (Figure 2A, left). The relative staining scores for all tissue samples showed a dramatic increase in ACL Ser-455 phosphorylation for the clinically invasive breast carcinomas (Figure 2A, right). ACL Ser-455 phosphorylation has been linked to AKT [26], and because mTORC2 directly activates AKT *via* Ser-473 phosphorylation, we next examined P-ACL(S455) and P-AKT(S473) profile in a panel of breast cancer cell lines. In a panel of 10 breast tumor lines, 6 cell lines (MDA361, MDA453, T47D, BT-474, BT-20, ZR-75-1) exhibited elevated and constitutive levels of P-ACL, while 3 cell lines (MDA231, HCC1806, MDA435) expressed low P-ACL levels and 1 line (MCF7) showed medium level of P-ACL that was repressed upon serum withdrawal (Figure 2B). The levels of P-ACL generally correlated well with the levels of P-AKT(S473) and all of the tumor lines with elevated P-ACL have well documented HER2-amplification (HER2+), mutational activation of PIK3CA (PIK3CAmut) or loss of PTEN. In a second HER2+/PIK3CAmut breast line MDA453, P-ACL was individually sensitive to mTOR inhibitor WYE-132, AKT inhibitor MK-2206, PI3K inhibitor GDC-0941 and HER2 inhibitor Lapatinib, supporting the notion that ACL Ser-455 phosphorylation is a common biomarker of the HER2/PIK3CA/mTOR/AKT network (Figure 2C). In this network, mTOR may act directly or through mTORC2-substrate AKT to phosphorylate ACL [26]. In Rat2 cells transformed with a constitutively active (membrane-bound) myr-AKT, both P-ACL and total ACL were robustly induced. Importantly, in this setting, both P-AKT(S473) and P-ACL(S455) were substantially reduced by WYE-132 but not by rapamycin, while the total ACL remained unchanged (Figure 2D). Collectively, these results reveal a critical role of mTOR in control of ACL phosphorylation.

### Phospho-ACL is a molecular target of mTORC2 and predicts breast cancer cell susceptibility to mTOR inhibition

**Figure 3:**
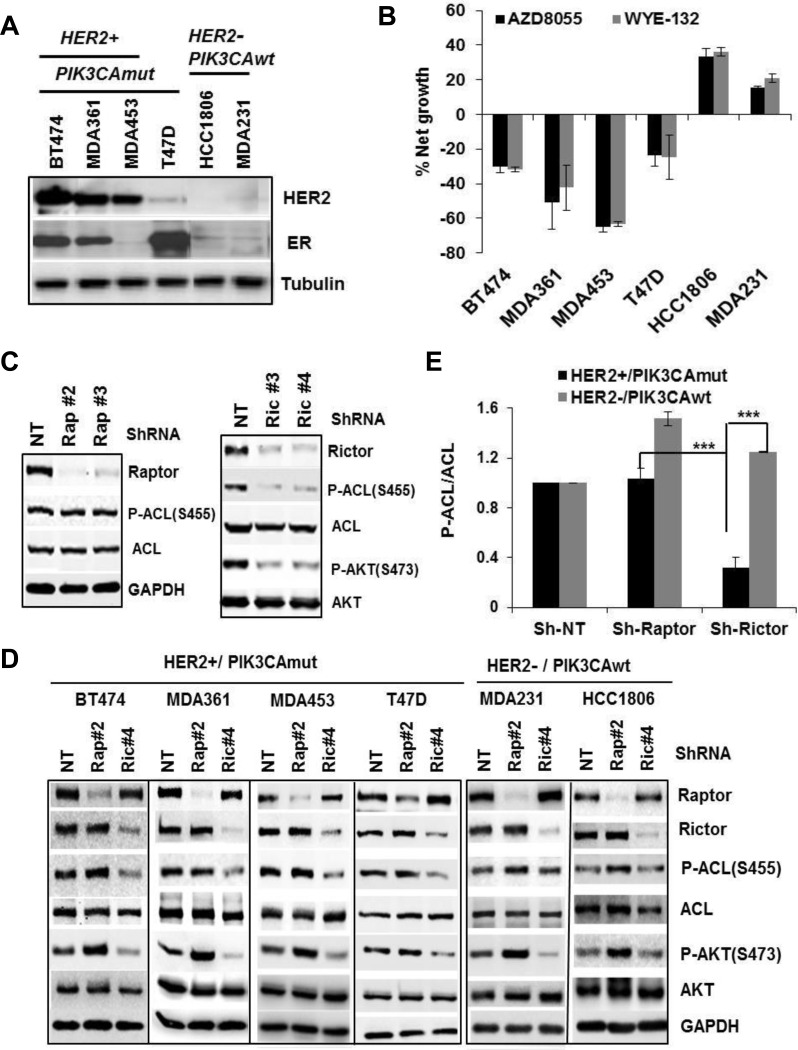
Phospho-ACL is a molecular target of mTORC2 **A.** Verification of HER2 and estrogen receptor (ER) status in 6 breast cell lines by immunoblotting. **B.** Cells were plated in 96-well plates for overnight, treated for 72 h with DMSO, 2 μmol/L WYE-132 or AZD8055, subjected to MTS assays. Net cell growth or death is relative to the initial cell density at initiation of inhibitor treatment. **C.** Validation of GIPZ-lentivirus shRNA against raptor- (Rap#2, Rap#3) or rictor (Ric#3, Ric#4) in MDA453 cells by immunoblotting. **D.** and **E.** Cells of 6 breast lines were infected with Sh-NT, Sh-Rap#2 or Sh-Ric#4, puromycin-resistant cell pools were selected and subjected to immunoblotting (D) and quantitated P-ACL/ACL levels are plotted (E). Statistical analysis: ***, *p* < 0.001.

If mTOR-ACL hyperactivation plays a role in HER2+/PIK3CAmut breast cancer cell survival, they might be particularly sensitive to mTOR-KI treatment. Indeed, treatment of HER2+/PIK3CAmut cells (BT-474, MDA361, MDA453 and T47D) with 2 μmol/L of two independent mTOR-KIs, WYE-132 or AZD8055, each caused a substantial cell death *via* apoptosis, whereas similar treatment of HER2-/PIK3CAwt cells (HCC1806 and MDA231) inhibited growth without cell death (Figure 3A, 3B, Figure S1B). This selective cell death was not due to variability in suppression of mTOR activity among these cells since both WYE-132 and AZD8055 invariably blocked P-AKT(S473) in all 6 cell lines (Figure S1B).

To clarify the relative contribution of mTOR complexes in ACL phosphorylation, we assessed effects of specific disruption of mTORC1 or mTORC2 both in HER2+/PIK3CAmut and HER2-/PIK3CAwt cells. After validating knockdown efficiency of various ShRNA hair pins against raptor, rictor or ACL, we chose two ShRNA from each gene set based on consistent performance in gene-depletion and functional testing (Figure S1C). Expression of two independent ShRNA for raptor or rictor yielded an efficient depletion of raptor or rictor protein leading to disruption of mTORC1 or mTORC2, respectively (Figure 3C). Importantly, in the HER2+/PIK3CAmut BT-474, MDA361, MDA453 and T47D cells, disruption of mTORC2 but not mTORC1 resulted in a substantial reduction in ACL Ser-455 phosphorylation (*p* < 0.001) without affecting total ACL, while similar depletion of mTORC2 in HER2-/PIK3CAwt MDA231 or HCC1806 cells did not reduce P-ACL (Figure 3D, 3E). A similar result was obtained using rictor- and raptor siRNA approach (Figure S1D). Notably, disruption of mTORC1 on average either had no effect or enhanced P-ACL levels (Figure 3D, Figure S1D). These results establish an essential role of mTORC2 in ACL Ser-455 phosphorylation in HER2+/PIK3CAmut breast cancer cells.

### mTORC2 but not mTORC1 is required for ACL-catalyzed acetyl-CoA production

**Figure 4:**
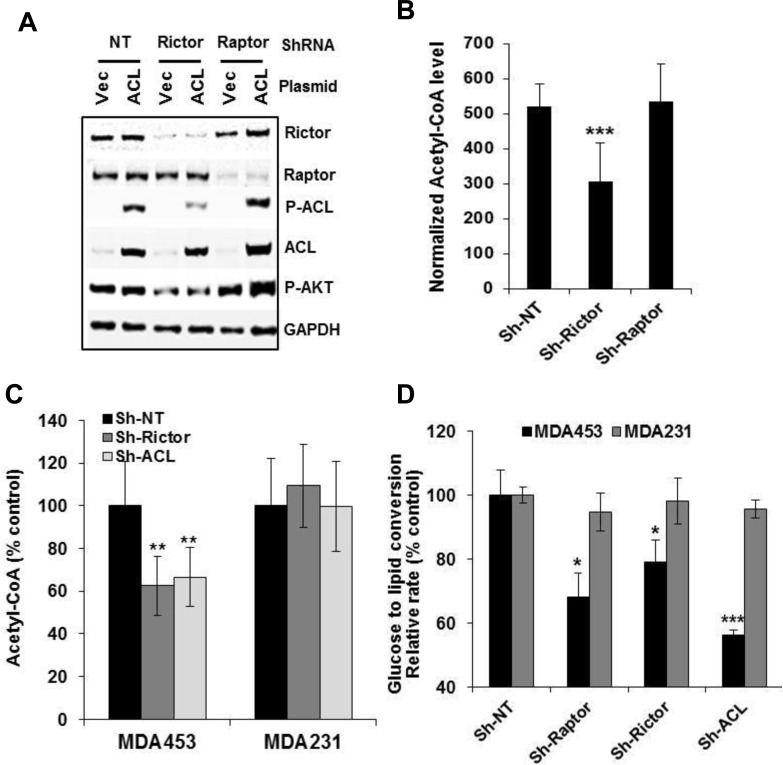
mTORC2 but not mTORC1 is required for ACL-catalyzed production of acetyl-CoA **A.** and **B.** 293T stable cell clones with doxycycline-inducible (pTRIPZ) lentiviral constructs for Sh-NT (control), Sh-Rap#2 or Sh-Ric#4 were induced with doxycycline for gene depletion. Cells were transfected with empty vector or ACL expression construct for 48 h, subjected to immunoblotting (A) and acetyl-CoA assay (B). ACL-dependent acetyl-CoA level (value of ACL-transfected normalized with the value of empty vector) obtained for each group are plotted. **C.** and **D.** MDA453 and MDA231 cells were infected with GIPZ-lentivirus encoding Sh-NT, Sh-Rap#2, Sh-Ric#4 or Sh-ACL#3. Puromycin-resistant cell pools were plated in 96-well plates for acetyl-CoA assay (C) or plated in 12-well plates, labeled with ^14^C-glucose for 2 h then subjected to measurement of total glucose-derived lipids as described in Methods. Statistical analysis: *, *p* < 0.05; **, *p* < 0.01; ***, *p* < 0.001.

Recent studies have shown that mTORC1 stimulates lipid synthesis through sterol responsive element binding proteins (SREBP1/2) [27, 28]. Given the importance of ACL in generating lipogenic precursor acetyl-CoA during glucose to lipid synthesis, we examined molecular specificity of the mTORC2-ACL connection and their role in acetyl-CoA production and glucose to lipid conversion (GTLC). A recombinant ACL was transfected into control-, raptor- or rictor-depleted 293T cells to study the specific ACL-dependent acetyl-CoA production. While ACL expression was robust in all cell populations (Figure 4A), depletion of rictor but not raptor substantially reduced the levels of P-ACL and ACL-catalyzed acetyl-CoA (*p* < 0.001) (Figure 4B), thus further confirming the molecular specificity and functional relevance of the mTORC2-ACL connection. We then measured cellular acetyl-CoA and glucose-derived lipids in rictor- or ACL-depleted MDA453 and MDA231 cells. Loss of mTORC2 or ACL each led to a significant reduction in level of acetyl-CoA (*p* < 0.01) in MDA453 cells (Figure 4C). Likewise, after labeling cells with ^14^C-glucose for 2 h, a significant reduction in glucose-derived lipid synthesis was observed in rictor-depleted (*p* < 0.05) or ACL-depleted (*p* < 0.01) and, as expected, in raptor-depleted (*p* < 0.05) MDA453 cells (Figure 4D). Notably, the reductions in acetyl-CoA and lipids were not apparent in gene-depleted MDA231 cells (Figure 4C, 4D), indicating a differential involvement of mTORC2-ACL in these two cell types.

### mTORC2-ACL broadly regulates de novo lipid synthesis and acetyl-CoA level in HER2/PIK3CA-hyperactive breast cancer cells

**Figure 5:**
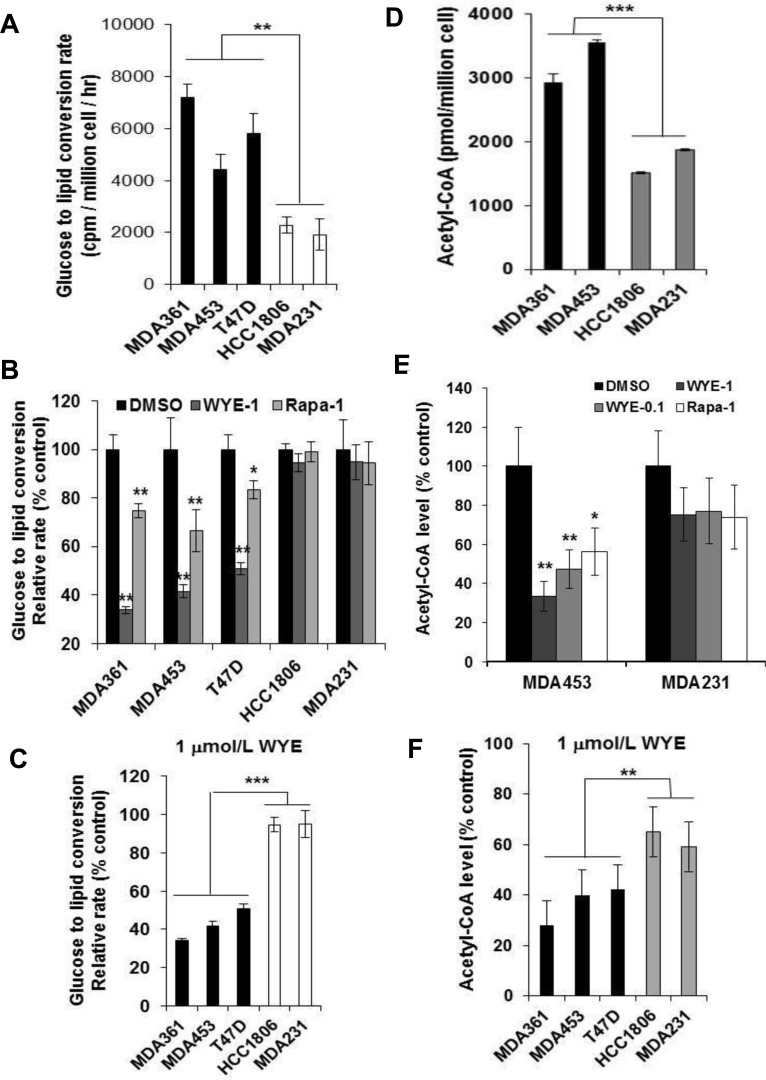
mTORC2-ACL regulates de novo lipogenesis in HER2/PIK3CA-hyperactive breast cancer cells **A.** Basal glucose to lipid conversion (GTLC) rates of 5 breast lines. Cells were cultured in 12-well plates, labeled with ^14^C-glucose for 2 h then measured for total glucose-derived lipids as described in Methods. **B.** Cells grown in 12-well plates were treated with DMSO, 1 μmol/L WYE-132 or rapamycin for 2 h, labeled with ^14^C-glucose and analyzed similarly as in A. **C.** Summary of relative GTLC rates in WYE-treated HER2+/PIK3CAmut MDA361, MDA453 and T47D cells *versus* HER2-/PIK3CAwt HCC1806 and MDA231 cells. **D.**-**F.** Measurement of basal acetyl-CoA level in exponentially proliferating HER2+/PIK3CAmut *versus* HER2-/PIK3CAwt cells (D) and relative levels of acetyl-CoA after 48 h treatment with 0.1, 1 μmol/L WYE-132 or 1 μmol/L rapamycin (E, F). Statistical analysis: *, *p* < 0.05; **, *p* < 0.01; ***, *p* < 0.001.

We found that the basal GTLC rates on average were significantly higher in MDA361, MDA453 and T47D cells than in HCC1806 and MDA231 cells (*p* < 0.01) (Figure 5A), implicating an involvement of high mTORC2-ACL activity in de novo lipid biosynthesis in HER2+/PIK3CAmut cells. We then measured GTLC rates after treatment of cells with DMSO, 1 μmol/L WYE-132 or rapamycin for 4 h. The glucose-derived total lipid production was profoundly inhibited in WYE-132-treated MDA361, MDA453 and T47D cells but were minimally inhibited in identically treated HCC1806 and MDA231 cells (*p* < 0.001) (Figure 5B, 5C). For comparison, rapamycin caused a significant but much smaller reduction in lipids (Figure 5B), which is consistent with the role of mTORC1 (24, 25). We next measured total acetyl-CoA level in cells treated without or with 1 μmol/L WYE-132 or rapamycin for 48 h. In exponentially proliferating state, the basal acetyl-CoA level was higher in MDA361 and MDA453 cells compared to that of HCC1806 and MDA231 cells (*p* < 0.001) (Figure 5D). While both WYE-132 and rapamycin were effective in reducing acetyl-CoA, WYE-132 caused a greater reduction (Figure 5E), and its inhibition of acetyl-CoA was more pronounced in MDA361, MDA453 and T47D cells compared to that of HCC1806 and MDA231 cells (*p* < 0.01) (Figure 5F). The results in Figure 4 and Figure 5 collectively indicate that in HER2+/PIK3CAmut cells the mTORC2-ACL axis promotes an enhanced lipogenic metabolism, which is efficiently reduced by pharmacological targeting of mTORC1 and mTORC2.

### mTORC2-ACL inhibition perturbs cell growth and mitochondrial physiology, which are partially rescued by an alternate source of acetyl-CoA

**Figure 6:**
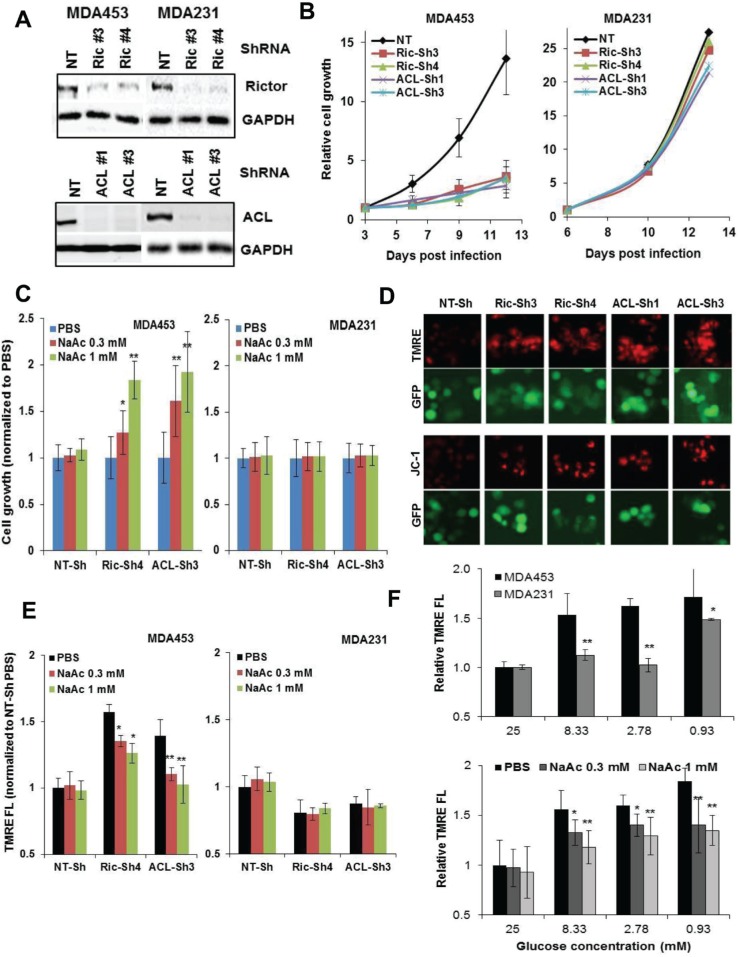
mTORC2-ACL inhibition perturbs cell growth and mΔψ, which involves homeostasis of glucose and acetyl-CoA **A.** and **B.** MDA453 and MDA231 cells were infected with GIPZ-lentivirus encoding Sh-NT, Sh-Ric#3 and Ric#4, or Sh-ACL#1 and ACL#3. Puromycin-resistant cell pools were cultured with puromycin and analyzed by immunoblotting (A) or growth measurement *via* cell counting (B). **C.** Cells as in B were analyzed for cell growth without or with NaAc supplement. **D.** MDA453 cells expressing the indicated ShRNA were cultured in 24-well plates then assessed for JC-1 or TMRE staining as described in Methods and subjected to fluorescence imaging. **E**. Cells as in B were plated in 96-well plates, cultured without or with NaAc supplement, stained with TMRE then monitored in a fluorescence plate reader. **F.** MDA453 and MDA231 cells (top panel) or MDA453 cells (bottom panel) were cultured in 24-well plates, fed fresh medium containing the indicated amounts of glucose without or with NaAC for 18 h, then subjected to TMRE fluorescence.

Cellular depletion of mTORC2 or ACL substantially reduced cell growth in MDA453 cells, while they minimally reduced growth in MDA231 cells (Figure 6A, 6B). The growth defect in MDA453 cells was partially but significantly rescued by an alternate source of acetyl-CoA *via* supplementing sodium acetate (NaAc) to culture medium (Figure 6C). It has been suggested that mTORC2 regulates mitochondrial physiology [29] and depletion of mTORC2 [29] or ACL [30] increased mitochondrial membrane potential (mΔψ). We found that treatment with 1 μmol/L WYE-132 selectively increased mΔψ in MDA453 but not MDA231 cells as measured by JC-1- or TMRE fluorescence (Figure S2A, S2B). This effect was recapitulated in mTORC2- or ACL-depleted MDA453 cells (Figure 6D, Figure S2C) and NaAc supplement resulted in a partial or nearly complete normalization of mΔψ in mTORC2- or ACL-depleted MDA453 cells, respectively (Figure 6E). In addition, a limited supply of glucose (2.8-8.3 mM) increased mΔψ in MDA453 but not in MDA231 cells, which was partially reverted by NaAc supplement (Figure 6F), indicating a differential metabolic regulation of glucose availability and mitochondrial homeostasis in these two cell types. Together, these results highlight a functional relationship between mTORC2-ACL activity and cell growth, which involves, at least in part, the homeostasis of glucose utilization, acetyl-CoA and mitochondrial physiology.

### mTOR directly phosphorylates ACL *in vitro* and Ser-455 phospho-mutants affect ACL function

**Figure 7:**
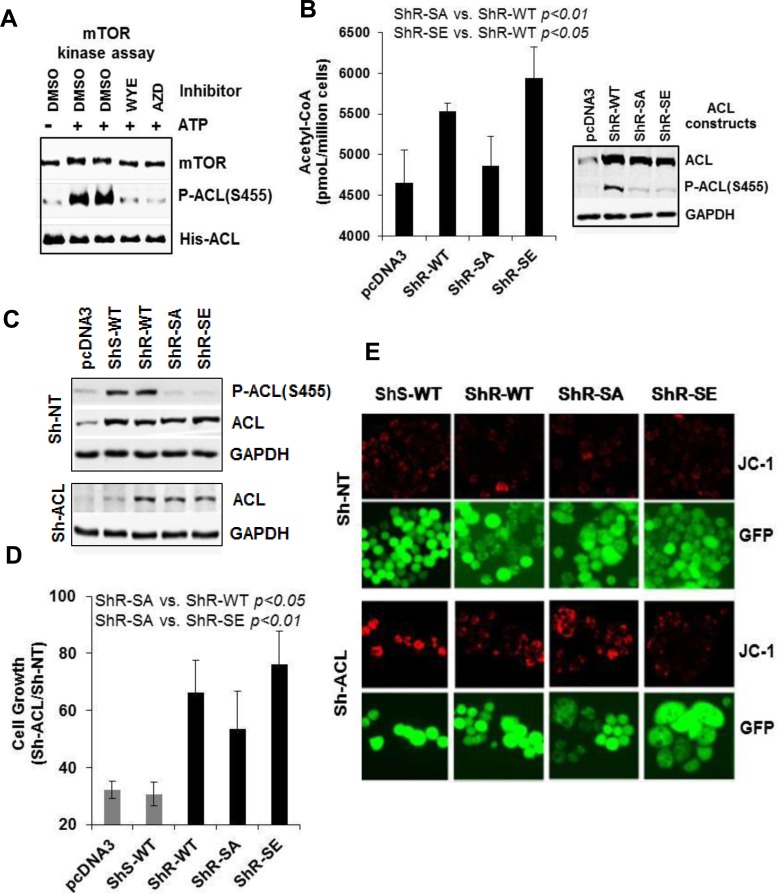
mTOR directly phosphorylates ACL and Ser-455 phospho-mutants affect ACL function **A.** mTOR kinase assay using ACL as a substrate as described in Methods. The reaction mixtures were immunoblotted. **B.** 293T cells were transiently transfected with the indicated ACL constructs for 48 h. The cells were assayed for cellular acetyl-CoA (left panel) or subjected to immunoblotting (right panel). **C.** MDA453 stable cell clones overexpressing the indicated ACL constructs were each infected with GIPZ-lentivirus encoding Sh-NT or Sh-ACL#3. Puromycin-resistant cells were subjected to immunoblotting. **D.** and **E.** Cells as in C were plated in 24-well plates and then assayed for cell growth by counting live GFP-positive cells (D) or assessed for JC-1 staining (E). For cell growth, values Sh-ACL relative to Sh-NT are shown. Statistical analyses are performed as indicated.

We considered the possibility that ACL could be a direct substrate of mTOR kinase activity. Employing a purified mTOR kinase domain capable of phosphorylating several known substrates of mTORC1 and mTORC2 [31], we performed *in vitro* kinase assay. mTOR efficiently phosphorylated a histidine-tagged ACL on Ser-455, which was completely inhibited by two selective mTOR-KIs WYE-132 or AZD8055 (Figure 7A). To further establish the functional relevance of Ser-455 phosphorylation, we prepared ShRNA-resistant wild-type ACL (ShR-WT) and then mutated its Ser-455 to alanine (ShR-SA, non-phosphorylatable) or glutamic acid (ShR-SE, phosphomimetic). In 293T cells, transient overexpression of wild-type ACL (ShR-WT) significantly increased cellular acetyl-CoA production compared to that of pcDNA3 vector control. Importantly, the alanine mutant ShR-SA showed a substantially reduced acetyl-CoA compared to that of ShR-WT (*p* < 0.01), while the glutamic acid mutant ShR-SE further increased acetyl-CoA relative to that of ShR-WT (*p* < 0.05) (Figure 7B). To assess the effects of ACL phospho-mutants in cell growth, MDA453 cells stably expressing various ACL constructs were infected with lentivirus encoding ACL-ShRNA or non-targeting NT-ShRNA control (Figure 7C). In pcDNA3- or ShRNA-sensitive ACL (ShS-ACL) expressing cells, depletion of endogenous ACL substantially reduced cell growth leading to 32% and 31% of control, respectively. Importantly, ShRNA-resistant ACL constructs rescued cell growth with ShR-SE, ShR-WT and ShR-SA achieving growth rates 76%, 66% and 54% of control, respectively (*p* < 0.05, ShR-WT *vs*. ShR-SA; *p* < 0.01, ShR-SE *vs*. ShR-SA) (Figure 7D). We also examined effects of ACL phospho-mutants on mΔψ *via* live JC-1 fluorescence. Upon depletion of endogenous ACL, ShS-WT cells exhibited a massive increase in JC-1 staining detected in nearly 100% of visualized cells (Figure 7E). The frequency and intensity of JC-1 staining were modestly changed in ShR-SA cells but were significantly reduced in ShR-WT cells and most efficiently eliminated in ShR-SE cells (Figure 7E). These results unequivocally establish a specific and critical role of mTORC2-dependent ACL Ser-455 phosphorylation in acetyl-CoA production, cell growth and mitochondrial homeostasis.

### Depletion of mTORC2 or ACL broadly inhibits HER2/PIK3CA-hyperactive tumor cell growth

**Figure 8:**
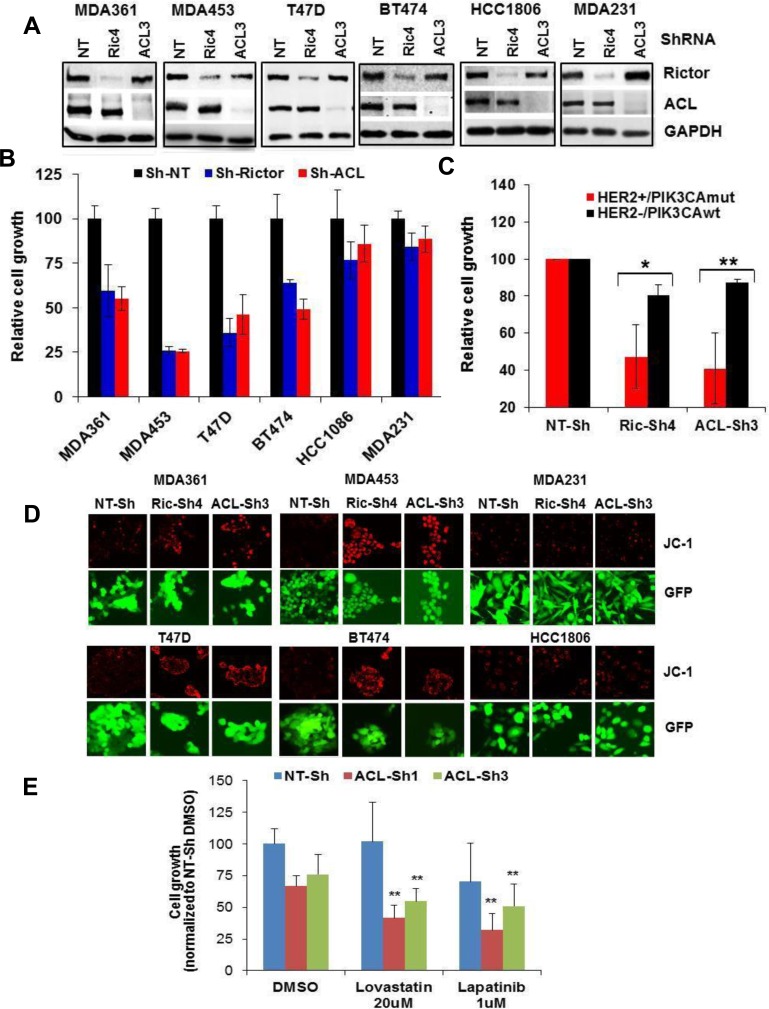
Depletion of mTORC2 or ACL broadly inhibits growth in HER2/PIK3CA-hyperactive breast tumor cells **A.** Cells of 6 breast lines were infected with GIPZ-lentivirus encoding Sh-NT, Sh-Ric#4 or Sh-ACL#3. Puromycin-resistant cell populations were selected and subjected to immunoblotting. **B.** Cells as in A were plated in 24-well plates and then assayed for cell growth by counting live GFP-positive cells. Cell growth values are relative to that of control Sh-NT. **C.** Mean values of HER2+/PIK3CAmut (MDA361, MDA453 and T47D) *versus* HER2-/PIK3CAwt (HCC1806 and MDA231) growth results in B are plotted. **D.** Cells as in A were grown in 24-well plates and subjected to live cell JC-1 staining. **E.** Cells as in A were plated in 96-well plates, treated with the inhibitors for 3 days, then assayed for cell growth as in B. Statistical analysis: *, *p* < 0.05; **, *p* < 0.01.

Finally, we determined the mTORC2- or ACL deficiency on growth and mΔψ in a panel of HER2+/PIK3CAmut *versus* HER2-/PIK3CAwt cells (Figure 8A). Significantly, while depletion of mTORC2 or ACL each reduced cell growth, the growth suppression on average was significantly greater in the HER2+/PIK3CAmut MDA361, MDA453, T47D and BT-474 cells compared to that of HER2-/PIK3CAwt HCC1806 and MDA231 cells (*p* < 0.05, Rictor-ShRNA cells; *p* < 0.01, ACL-ShRNA cells) (Figure 8B, 8C). Consistent with the previous results, depletion of mTORC2 or ACL correlated with a striking increase in JC-1 staining in all four HER2+/PIK3CAmut lines but not in the two HER2-/PIK3CAwt lines (Figure 8D). Lastly, depletion of ACL in MDA453 cells enhanced the growth-inhibitory effects by the lipid-lowing drug Lovastatin (*p* < 0.01) and by the HER2 kinase inhibitor Lapatinib (*p* < 0.01) (Figure 8E). Collectively, these results further support an important role for mTORC2-ACL axis in cell growth and therapeutic response in HER2/PIK3CA-hyperactive breast cancer.

## DISCUSSION

Successful development of mTOR-targeted cancer treatment will require molecular understanding of mTORC1 and mTORC2 in oncogenesis and therapy resistance. While mTORC1 has been a validated cancer drug target for years, the role of mTORC2 remains much less defined. More recent studies have suggested an essential role of mTORC2 in cellular transformation driven by oncogenic HER2 [32] or EGFRvIII [33]. In HER2-mutant breast cancer, mutational activation of the PI3K/AKT/mTOR pathway is among the most frequent mechanisms contributing to both the intrinsic and acquired therapy resistances [34–36]. A recent report demonstrated that mTORC2 can be directly activated by PtdIns(3,4,5)P3 through its binding to the PH-domain of Sin1, a component of mTORC2 [37]. Thus, mTORC2 acts as a major signaling hub to amplify oncogenic signals from receptor tyrosine kinases (RTKs) and/or PI3K, both upstream of and in parallel to mTORC1. Moreover, patient tumor derived-genetic alterations in mTORC2 component Sin1 [37] or Rictor [38] have been recently reported. Collectively, these earlier studies indicate a general role for mTORC2 in tumorigenesis, which is not efficiently targeted by the rapalog therapy.

In this report, we aimed to identify new role of mTORC2 in breast cancer. Through a phosphoproteomic analysis and subsequent characterization, we discovered Ser-455 of ACL as a molecular target of mTORC2. Elevated ACL Ser-455 phosphorylation correlated well with stage and invasiveness of human breast tumors and with HER2+/PIK3CAmut status in tumor cell lines. Depletion of mTORC2 but not mTORC1 inhibited ACL Ser-455 phosphorylation in the HER2+/PIK3CAmut BT-474, MDA361, MDA453 and T47D cells, while similar depletion of mTORC2 did not diminish phospho-ACL in HER2-/PIK3CAwt MDA231 and HCC1806 cells. Importantly, cells expressing high phospho-ACL were generally more susceptible to growth inhibition targeted by mTOR-KIs, mTORC2-depletion or ACL-depletion. Biochemical studies indicated that mTOR directly and specifically phosphorylated ACL on Ser-455 *in vitro*. While this observation is interesting, it is unknown whether mTORC2 directly phosphorylates ACL *in vivo*. The observation that both WYE-132 and MK-2066 inhibited phospho-ACL is consistent with the notion that mTORC2 signals, at least in part, through AKT to phosphorylate ACL [26]. Our analysis of ACL Ser-455 mutants encoding a non-phosphorylatable S455A or phosphomimetic S455E further confirmed the functional importance of Ser-455 phosphorylation in acetyl-CoA production, mitochondrial homeostasis and cell growth. Thus, these observations established an important role for mTORC2-dependent ACL phosphorylation in HER2+/PIK3CAmut breast cancer cells.

Our results along with earlier studies highlighted a role of mTOR in lipid synthesis in tumorigenesis. Previous work have suggested mTORC1 as an activator of SREBP1/2 in lipogenesis in AKT-transformed cells [27] or breast tumor cells [28]. In the present study, we uncovered mTORC2-ACL as a positive regulator of lipogenesis. The IGF-1-induced phospho-ACL and de novo lipid synthesis were inhibited by mTOR-KI but not by rapamycin, and depletion of mTORC2 but not mTORC1 specifically reduced the ACL-dependent acetyl-CoA production. In HER2+/PIK3CAmut breast tumor cells, an elevated mTORC2-ACL activity is prevalent and correlates with higher basal levels of acetyl-CoA and de novo lipid synthesis, both of which were more substantially inhibited by mTOR-KI than rapamycin. In contrast, mTOR inhibition only modestly reduced de novo lipid synthesis in HER2-/PIK3CAwt cells. These findings suggest that the mTORC2-ACL-dependent lipogenic reprograming is more important in supporting the HER2/PIK3CA-driven tumor cell growth.

Perturbation of cytosolic acetyl-CoA level is relevant for antitumor activity in mTORC2-ACL inhibitor therapy. Aside from its role in lipogenesis, acetyl-CoA is the sole acetyl- group donor for protein acetylation, thereby affecting diverse cellular functions of metabolism, autophagy, signal transduction and transcriptional control [39, 40]. ACL positively regulates histone acetylation [41] linking acetyl-CoA availability with cell growth and proliferation [42, 43]. In our hands, mTOR pharmacological inhibitor, genetic inactivation of mTORC2 or ACL each induced a more severe decline in acetyl-CoA level in a panel of HER2+/PIK3CAmut cells compared to that of HER2-/PIK3CAwt cells, correlating a dramatic growth suppression and mitochondrial hyperpolarization in these cells. Importantly, an alternate source of acetyl-CoA *via* NaAc partially rescued growth defects and mitochondrial hyperpolarization induced by mTORC2/ACL-depletion in MDA453 cells. In contrast, NaAc had little effect in mTORC2/ACL-depleted MDA231 cells. These observations suggested an important role for mTORC2-ACL in acetyl-CoA homeostasis and, presumably, the acetyl-CoA-sensitive functions in HER2/PIK3CA-hyperactive breast cancer cells. Lastly, consistent with previous studies [30, 44–47], our results also highlighted ACL as a functional biomarker in breast tumorigenesis and a potential target for drug development.

In summary, we have identified a novel and critical role of mTORC2 in oncogenic mechanism of HER2/PIK3CA-hyperactive breast cancer. mTORC2 phosphorylates and activates ACL thereby promoting an elevated de novo lipogenesis and acetyl-CoA function. This “reprogrammed” metabolic state is highly susceptible to mTOR-KI treatment and may predict therapeutic efficacy in cancer patients.

## MATERIALS AND METHODS

### Chemicals, RNA interference and plasmids

WYE-125132, rapamycin and PI-103 were either purchased from BiochemPartner (Shanghai) for work at Fudan University or obtained from Discovery Chemistry for work at Pfizer (formerly Wyeth). AZD8055, MK-2066, GDC-0941 and Lapatinib were purchased from BiochemPartner (Shanghai). Recombinant human IGF-1 was purchased from R&D system. All other chemicals were purchased from Sigma-Aldrich. Inhibitors were dissolved in DMSO as 20 mM stock solution and were diluted before assays. GIPZ shRNA sets (3-4 hairpins per gene) for human Raptor (Cat#RHS4531-EG57521), Rictor (Cat#RHS4531-EG253260), ACL (Cat#RHS4531-EG47) and non-targeting (NT, Cat#RHS4346) were obtained from Open Biosystems. ON-TARGET plus siRNA pools of non-targeting control, Raptor and Rictor were obtained from Dharmacon-ThermoScientific. A full-length human ACL cDNA (Genebank#NM_001096.2) was obtained from Sino Biological (Beijing) and cloned into pcDNA3.1+, defined as ShS-WT. An ShRNA#3-resistant version of ACL was created by overlapping PCR, defined as ShR-WT. ShR-WT was used to generate S455A (ShR-SA) or S455E (ShR-SE) *via* Quik-Change mutagenesis kit (Stratagene). The myr-AKT construct has been described [48].

### mTOR kinase assay of ACL

mTOR kinase assay was performed with recombinant mTOR enzyme (Millipore, Cat#14-770) and unphosphorylated ACL protein (Sino Biological, Cat#11769-H07B) following the method described [8].

### Cell culture, gene knockdown, gene transfection, assays of cell survival and mitochondrial membrane potential

Cell lines used in this study were obtained from ATCC unless otherwise noted. Cells were cultured in a 37°C incubator with 5% CO_2_ using standard cell culture methods and reagents (Invitrogen). To create gene depletion, various pGIPZ-ShRNA constructs (or equivalent pTRIPZ format) were packaged in 293T cells and validated according to manufacturer's instruction. Breast cells were infected GIPZ or TRIPZ virus, selected by puromycin for stable cell population. ON-TARGET SiRNA pools were transfected to cells using lipofecatmine 2000 reagent (Invitrogen) and analyzed 72 h later. To express various ACL constructs, plasmid DNA cloned into pcDNA3.1+ was transfected into the indicated cells and, in some studies, selected by G418 for stable expression population. Cell survival assays were conducted in 96-well plate or 24-well plate at 10-20% confluence with inhibitor treatment for 3 days. Viable cells were counted by trypan blue exclusion method or by MTS dye converting assay using CellTiter AQ assay kit (Promega). Viability and growth of various GIPZ-ShRNA-expressing cells was determined by counting puromycin-resistant GFP-positive cells. Mitochondrial membrane potential was assessed using 5,5′,6,6′-tetrachloro-1,1′,3,3′-tetraethylbenzimidazole carbocyanide iodide (JC-1) (Cat#22200, AAT Bioquest) or tetramethylrhodamine ethyl ester (TMRE, Cat#87917), carbonyl cyanide m-chlorophenylhydrazone (CCCP, Cat#C2759) obtained from Sigma-Aldrich. Cells with various treatments were stained with 3 μg/mL JC-1 or 1 μM TMRE in serum-free medium at 37°C for 15 to 30 min, washed and monitored under a fluorescence microscope or by a fluorescence plate reader.

### Immunoblotting, immunohistochemistry and immunofluorescence

After various treatments cells were lysed using NuPAGE-LDS sample buffer (Invitrogen). For profile of breast cancer panel, cells were lysed as described [49]. Total cell lysates were immunoblotted with antibodies P-ACL(S455) (Cat#4331), ACL (Cat#4332), HER2 (Cat#2242) and estrogen receptor (Cat#8644) (Cell Signaling Technology). Immunoblotting of other proteins were performed as described previously [50]. Breast tissue arrays were obtained from Pantomics (Cat#BRD181) and Biomax US (Cat#T081). Immunohistochemistry was performed with rabbit anti-P-ACL(S455) (Abcam, Cat#59297), anti-rabbit Ig reagents were developed using 3,3′-diaminobenzidine chromogen (DAKO). Images were taken using Olympus BX61 microscope with MicroSuite Pathology Addition software. For immunofluorescence, cells seeded on collagen coated cover slips and treated with mTOR inhibitor for 16 h were stained with the same rabbit anti-P-ACL (S455), Alexa Fluor 594 donkey anti-rabbit IgG (Invitrogen, Cat#21207). Images were acquired using Carl Zeiss LSM 510 Meta confocal leaser scanning microscope.

### Assays of de novo lipid synthesis and acetyl-CoA

For de novo lipid synthesis, cells were grown in 12-well plates to ~50% confluence, re-fed with medium RPMI-1640 with 1% FBS for overnight. Cells were pretreated with inhibitors for 2 h, and then labeled for 2 h with 2 μCi/ml of D-[1-14C] glucose (Perkin Elmer, Cat#NEC043X001MC). Total lipids were extracted twice with 300 μL each of hexane:isopropanol (3:2; vol:vol) as described [30]. Cell debris was carefully removed by centrifugation at 14,000 rpm for 5 min using a microcentrifuge. Radioactive lipids were dried, re-dissolved with 100 μL of chloroform and counted in 5 mL scintillation cocktail. A duplicate assay plate without radioactive glucose label was used to count the number of cells per treatment. Glucose to total lipid conversion rate is the radioactive counts from the extracted lipid normalized by the number of cells per hour of ^14^C-glucose incubation. For acetyl-CoA assay, cells grown in 96-well culture plates were washed and lysed in 40 μL/well using lysis buffer described [49]. Cell lysates were transferred to V-bottom plates, cleared by centrifugation. 30 μL of cleared cell lysates were subjected to acetyl-CoA measurement using an acetyl-CoA assay kit (Sigma, Cat#MAK-039) following manufacturer's assay manual. Duplicate assay plates were used to count the number of cells per treatment for normalization in determining cellular acetyl-CoA levels.

### Stable isotope labeling by amino acids in cell culture (SILAC)-based phosphoproteomics

Detailed method for MDA361 cell SILAC study has been described previously [51].

## SUPPLEMENTARY MATERIAL FIGURES


