# Supplementary material for: mTOR complex-2 stimulates acetyl-CoA and de novo lipogenesis through ATP citrate lyase in HER2/PIK3CA-hyperactive breast cancer
Source: Oncotarget. 2016 Mar 22;7(18):25224–40. doi: 10.18632/oncotarget.8279 (PMC5041899; doi:10.18632/oncotarget.8279)
Supplement: Supplementary file 1 [file oncotarget-07-25224-s001.pdf]

mTOR complex-2 stimulates acetyl-CoA and *de novo* lipogenesis through ATP citrate lyase in HER2/PIK3CA-hyperactive breast cancer

Supplementary Material

Figure S1

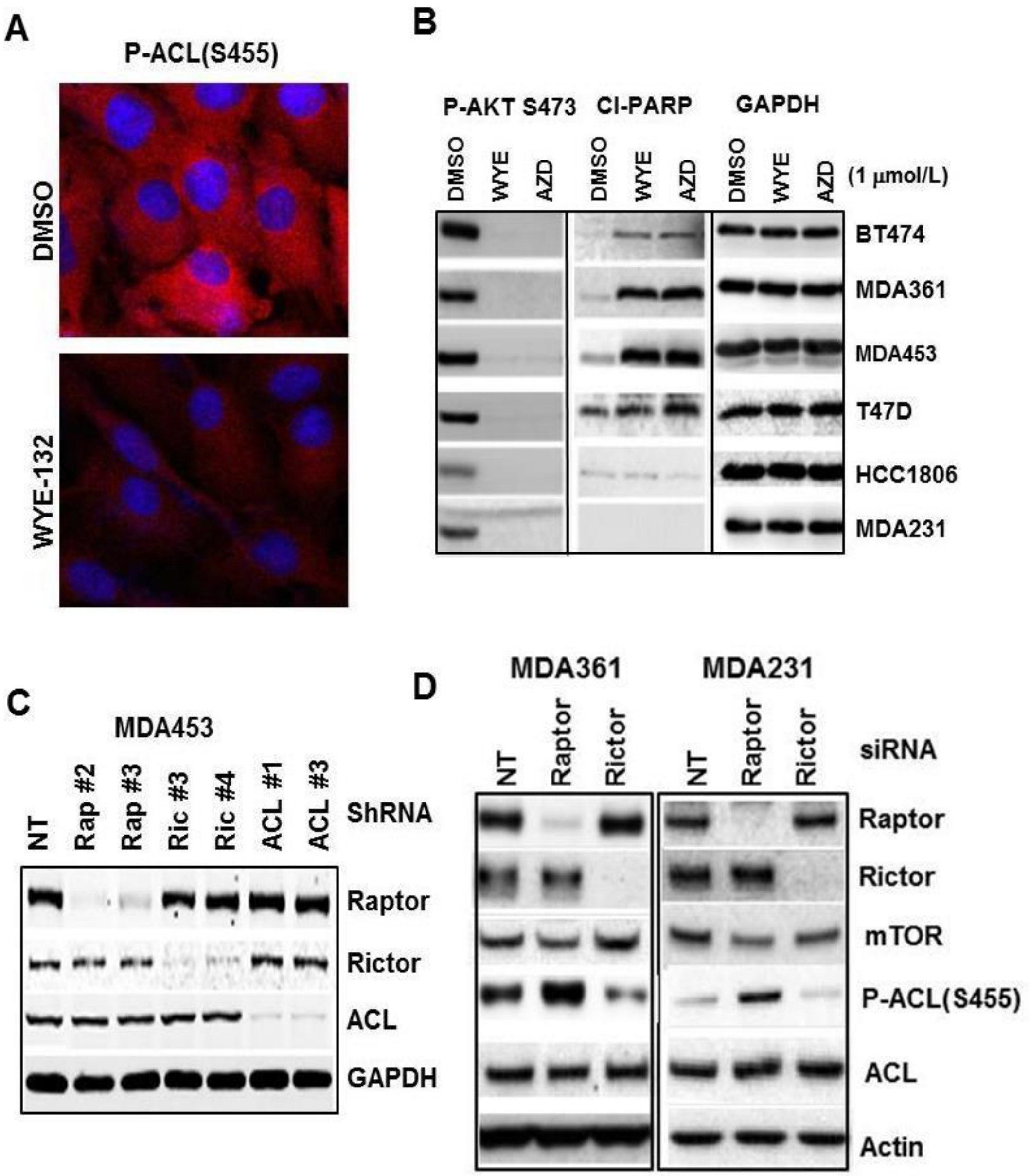

Figure S1. **A.** Immunofluorescence of ACL phospho-Ser-455 in A498 cells. Cells seeded on collagen coated cover slips and treated with DMSO or 1 μmol/L WYE-132 for 16 h were stained with anti-P-ACL (S455) as described in Method. **B.** Differential cell survival in response to mTOR kinase inhibitor. The indicated cells were plated in 6-well plate, treated with 1 μmol/L WYE-132 or AZD8055 for 48 h then subjected to immunoblotting. **C.** Validation of pGIPZ-based lentiviral ShRNAs against raptor, rictor and ACL. 3-5 hair pins per target were packaged in 293T cells. MDA453 cells were infected with lentivirus, selected with puromycin, analyzed by immunoblotting. The validation studies identified 2 hair pins for each target gene. Gene knockdown efficiency of Sh-Rap#2 and Rap#3, Sh-Ric#3 and Ric#4, Sh-ACL#1 and ACL#3 are shown; the selected ShRNA pairs are routinely used with consistent profile in various functional testings. **D.** Depletion of mTORC2 but not mTORC1 inhibited phospho-ACL in MDA361 but not MDA231 cells. Cells were transfected with Raptor- or Rictor SiRNA for 72 h. Total cell lysates were prepared and subjected to immunoblotting.

Figure S2

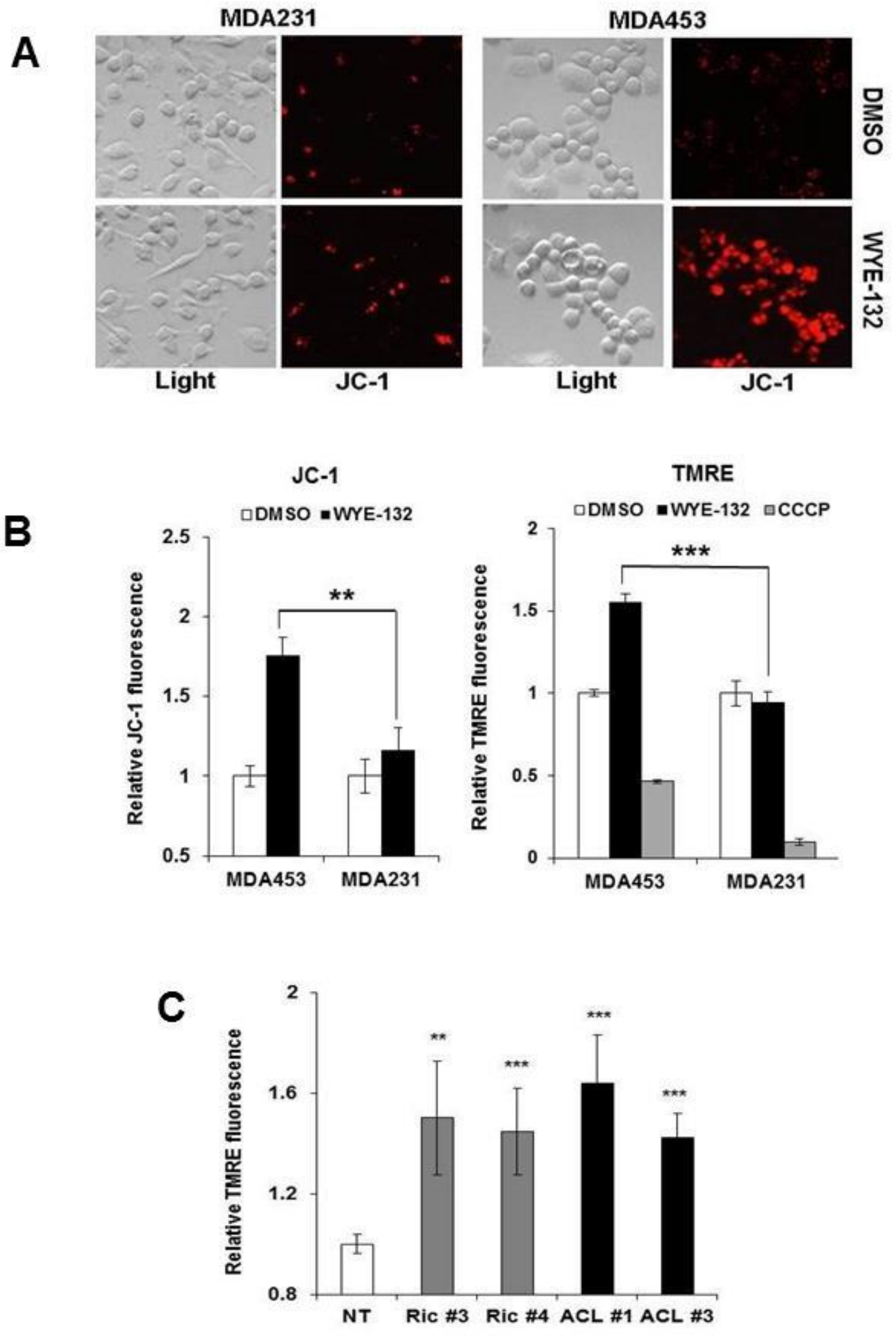
A
MDA231
MDA453
DMSO
WYE-132
Light
JC-1
Light
JC-1
B
JC-1
□DMSO ■WYE-132
Relative JC-1 fluorescence
2.5
2
1.5
1
0.5
**
MDA453
MDA231
TMRE
□DMSO ■WYE-132 ■CCCP
Relative TMRE fluorescence
2
1.5
1
0.5
0
***
MDA453
MDA231
C
Relative TMRE fluorescence
2
1.6
1.2
0.8
**
***
***
***
NT
Ric #3
Ric #4
ACL #1
ACL #3

Figure S2. Targeting of mTORC2-ACL increases mitochondrial membrane potential (m$\Delta\psi$). **A.** MDA231 and MDA453 cells were treated with DMSO or 1 μmol/L WYE-132 for 16 h, stained with JC-1 and subjected to fluorescence imaging. **B.** Cells treated as in A were stained with JC-1 and TMRE then monitored by a fluorescence plate reader. **C.** Cells expressing the indicated GIPZ-ShRNAs were grown in identical culture medium, stained with TMRE then monitored by a fluorescence plate reader. The values were normalized to cell number.
